# Treatment Trials for Neonatal Seizures: The Effect of Design on Sample Size

**DOI:** 10.1371/journal.pone.0165693

**Published:** 2016-11-08

**Authors:** Nathan J. Stevenson, Geraldine B. Boylan, Lena Hellström-Westas, Sampsa Vanhatalo

**Affiliations:** 1 Irish Centre for Fetal and Neonatal Translational Research and Department of Paediatrics and Child Health, University College Cork, Cork, Ireland; 2 Department of Women's and Children's Health, Uppsala University, Uppsala, Sweden; 3 Department of Clinical Neurophysiology HUS Medical Imaging Center, Helsinki University Central Hospital, Helsinki, Finland; 4 Department of Neurological Sciences, Clinicum, University of Helsinki, Helsinki, Finland; University of Modena and Reggio Emilia, ITALY

## Abstract

Neonatal seizures are common in the neonatal intensive care unit. Clinicians treat these seizures with several anti-epileptic drugs (AEDs) to reduce seizures in a neonate. Current AEDs exhibit sub-optimal efficacy and several randomized control trials (RCT) of novel AEDs are planned. The aim of this study was to measure the influence of trial design on the required sample size of a RCT. We used seizure time courses from 41 term neonates with hypoxic ischaemic encephalopathy to build seizure treatment trial simulations. We used five outcome measures, three AED protocols, eight treatment delays from seizure onset (T_d_) and four levels of trial AED efficacy to simulate different RCTs. We performed power calculations for each RCT design and analysed the resultant sample size. We also assessed the rate of false positives, or placebo effect, in typical uncontrolled studies. We found that the false positive rate ranged from 5 to 85% of patients depending on RCT design. For controlled trials, the choice of outcome measure had the largest effect on sample size with median differences of 30.7 fold (IQR: 13.7–40.0) across a range of AED protocols, T_d_ and trial AED efficacy (p<0.001). RCTs that compared the trial AED with positive controls required sample sizes with a median fold increase of 3.2 (IQR: 1.9–11.9; p<0.001). Delays in AED administration from seizure onset also increased the required sample size 2.1 fold (IQR: 1.7–2.9; p<0.001). Subgroup analysis showed that RCTs in neonates treated with hypothermia required a median fold increase in sample size of 2.6 (IQR: 2.4–3.0) compared to trials in normothermic neonates (p<0.001). These results show that RCT design has a profound influence on the required sample size. Trials that use a control group, appropriate outcome measure, and control for differences in T_d_ between groups in analysis will be valid and minimise sample size.

## Introduction

Evidence-based guidelines of drug treatments require studies where the drug effect is quantitatively measured and statistically compared to an alternative treatment or placebo. Key elements of successful trial designs include the choice of relevant outcome measure(s) and sample size. Selection of the outcome measure is typically a compromise between what is known to be important in pathophysiology and what is practically possible. While the sample size must be selected so that it is large enough to demonstrate statistical significance of a clinically relevant effect, there is a practical need to minimize the sample size via trial design in order to reduce the cost and duration of trials in vulnerable patient groups with rare conditions. The details of RCT design are, therefore, critical when interpreting study findings; particularly when study findings lead to a change in clinical practice.

Measuring treatment outcome is challenging when the natural course of the illness is variable and has a tendency to improve [1, 2]. It becomes even more difficult when the natural duration of the illness is short relative to the timing of treatment protocols [3]. Many neurological illnesses, such as status epilepticus, and migraine, are well known to have highly variable and self-limiting time courses. This is further complicated by the fact that the partial effectiveness of existing medications (such as phenobarbitone) may preclude the use of a placebo resulting instead in the use of a positive control group [4, 5].

Seizures in neonates present a particular challenge in this context because seizures tend to resolve within tens of hours, leaving little time to observe treatment effects [6, 7]. In addition, seizure occurrence is highly variable across neonates and varies with aetiology [7–10]. It is generally accepted that the accumulated duration of seizures, or seizure burden, as measured by multi-channel EEG, should be the quantitative measure of choice when assessing anti-epileptic drug (AED) efficacy [4, 5, 11–13]. This is based on the assumption that a high seizure burden causes further damage to the already compromised neonatal brain [14–16]. An evidence based AED trial should, therefore, be powered so that it takes into account the natural time course of neonatal seizures. Otherwise, a bias in outcome towards a positive treatment effect could be introduced. It has been difficult to accurately power a neonatal AED trial *a priori*, due to limited data on the temporal behaviour of seizures over a period of days. Recent advances in long term EEG monitoring have shed light onto the temporal evolution of neonatal seizures which will improve power calculations [7, 10].

In the present work, we aimed to establish the effect of different designs of neonatal AED trials on the sample size estimate from a power analysis. To this end, we modelled seizure time courses based on real data, which allowed us to examine the influence of trial design on the sample size for different outcome measures, AED protocol and delays in intervention at different levels of AED efficacy.

## Methods

This study was performed as a series of simulated AED trials for neonatal seizures which allows direct comparison between different trial designs. The key prior knowledge required for realistic simulations is the natural time course of seizure burden during neonatal seizures. Here, we took advantage of two recently collected cohorts of long-term EEG recordings from neonates with seizures due to hypoxic ischemic encephalopathy (HIE) [7, 10]. These cohorts contained complete, second by second, quantification of seizure burden in two cohorts of neonates collected before (n = 18, normothermic group) and after (n = 23) the introduction of hypothermia as treatment for HIE [7, 10]. In these studies, data collection was conducted with approval from the Clinical Research Ethics Committee of the Cork Teaching Hospitals, Ireland. Parental written, informed consent was obtained for all newborns recruited for EEG monitoring studies. All data were anonymised. For more details on the demographics and seizure burden of this cohort see the S1 Appendix and Lynch et al. (2012 and 2014) [7, 10]. The most significant difference between these two cohorts was a lower overall seizure burden in neonates treated with hypothermia.

### Generation of a realistic model of neonatal seizure burden time courses

We first constructed a realistic model of seizure burden time courses that permitted the use of large simulated cohorts. A lognormal function was found to adequately simulate the seizure burden time course with the characteristic positive skewness that is seen in the distribution of seizures over time. In other words, neonatal seizure burden has been shown to accumulate rapidly in the hours after seizure onset, followed by a more gradual accumulation towards seizure offset [7]. It also results in smoothed time courses where seizures occur to some extent for the entire period of simulation. The smoothing of any discontinuities in the seizure burden time courses was required for the following reasons: 1) discontinuities generated by the response to AED treatment and periods of missing data contaminate the normative seizure burden time course resulting in the need for interpolation, 2) the systematic implementation of the RCT simulations would result in invalid outcome measures in the presence of discontinuity, i.e. no seizure burden (further, an AED would not be given if no seizures were present), 3) the function of seizure burden over time is not a raw value and must be calculated from the raw seizure annotations, so smoothing the seizure burden can be considered as calculating the seizure burden over a longer time period (see S1 Appendix). Notably, while smoothing may reduce the peak seizure burden, it does not significantly alter other important summary measures of seizure burden such as the total seizure burden, the skew towards seizure onset, and the time from seizure onset to the point of maximum seizure burden.

**Fig 1:**
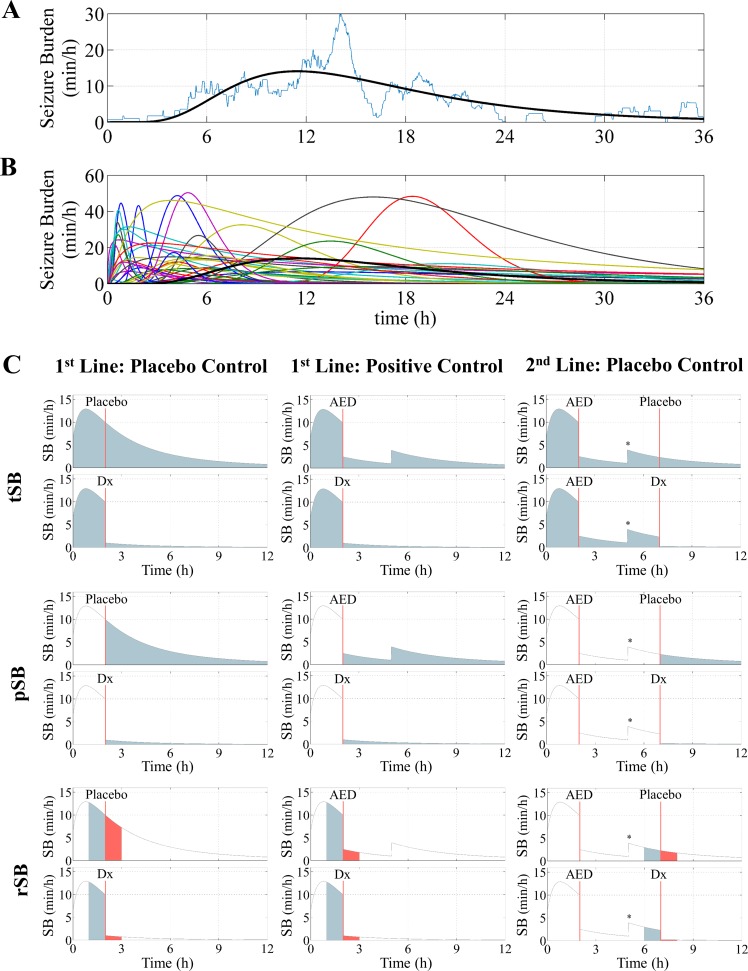
Seizure time courses and neonatal seizure treatment trial designs. The simulated seizure time courses used to estimate the sample size for various designs of a randomized control trial. A) An example smoothed seizure time course from the cohort of Lynch et al. (2015) plotted over the corresponding real seizure time course [10]. B) All 41 smoothed seizure time courses from the cohorts of Lynch et al. (2012 and 2015) showing the variability of seizures in neonates (the black line in A and B refers to the same neonate) [7, 10]. SB/h is seizure burden in minutes per hour; it is a measure of the short term intensity of seizures. Time is measured with respect to seizure onset. C) The use of different AED protocols and outcome measures in RCT design. Each row defines a common outcome measure and each column defines an AED protocol. Outcome measures are defined by the shaded areas: tSB row–total SB (blue shaded area), pSB row–post-intervention SB (blue shaded area), and rSB row–SB response (blue shaded area subtracted from the red shaded area). Treatment delay is the difference between seizure onset and the initiation of the trial protocol and is 2h in these examples. The level of AED efficacy in these examples is an immediate 90% reduction in seizure burden. The trial drug is denoted as Dx. Existing or positive control anti-epileptic drug (AED) effect was based on phenobarbitone: a 75% reduction in seizure burden for 3h. The asterisk denotes the cessation of the existing AED effect (seizure reoccurrence).

In order to generate a single seizure burden time course, the parameters of the lognormal function were assumed to be random and were selected from a multi-variate distribution estimated from real data. An example of the lognormal function fitted to real data and the lognormal function fitted to all 41 neonates in the cohort are shown in Fig 1A and 1B. More details on the process of simulating seizure burden time courses and the quality of fit of a lognormal function to real data is outlined in S2 Appendix.

### Simulation of therapeutic trials

The simulated seizure burden time courses were used in a power calculation for several randomized control trials (RCT). Each RCT was defined using several variables: AED protocol, average time relating to the administration of the intervention with respect to seizure onset (T_d_), target level of trial AED efficacy and outcome measure.

Three different AED protocols were used (see Fig 1C): first line AED vs. placebo control, first line AED vs. positive control (assumed to be phenobarbitone), and second line AED vs. placebo control (with phenobarbitone as a first line). The efficacy of phenobarbitone was assumed to be a 75% reduction in seizures for 3h [17]. The T_d_ variable represents delays in the clinical recognition of seizures and the speed of execution of the trial protocol and was varied from 1h to 8h [18]. We used four AED efficacies from a maximum possible effect (100% reduction in seizures for 72h), to more typical effects (80% reduction for 12h, 80% reduction for 6h, and 50% reduction for 12h) that parallel typical target levels of efficacy [4, 12, 13].

Five outcome measures were estimated from the simulated seizure burden time course (for a graphical representation, see Fig 1C). 1) Total seizure burden: The total accumulated duration of seizures between seizure onset and seizure offset (or monitoring cessation). 2) Post-intervention seizure burden for 1h: The accumulated duration of seizures in a 1h time period after the intervention. 3) Post-intervention seizure burden for 12h: The accumulated duration of seizures in a 12h time period after the intervention. 4) Seizure burden response over 1h: The accumulated duration of seizures in a one-hour time period before the intervention (baseline) subtracted from the accumulated duration of seizures in a 1h time period after the intervention. 5) Seizure burden response over 12h: The accumulated duration of seizures in a one-hour baseline period subtracted from the accumulated duration of seizures averaged across a 12h time period after the intervention.

The total seizure burden is the most general measure of seizures in a neonate and maximising its reduction should have the most positive effect on long term neurodevelopmental outcomes [15]. This outcome measure requires long term, continuous EEG recording from the onset of seizures, which may be logistically challenging in many neonatal intensive care units (NICUs). The post-intervention seizure burden takes into account the seizure burden after the intervention in each patient while the seizure burden before the intervention is ignored. This outcome measure requires a continuous EEG recording from the time of intervention, which is achievable in most NICUs. The seizure burden response is commonly used in uncontrolled studies and requires EEG recording during the pre- and post-intervention time periods.

Several published trials use the seizure burden response as the primary outcome measure [4, 5, 12, 13]. The advantage of the seizure burden response is that it is the only outcome measure that can be used in an uncontrolled study. The total seizure burden has been used to assess treatments such as hypothermia and the usefulness of EEG monitoring to guide treatment [16, 19].

### Estimating the sample size (power calculation)

The sample size was calculated using the mean and standard deviations of these outcome measures in simulated arms of the RCT. The sample size was defined as,

$$N=\frac{4(\sigma_{1}^{2}+\sigma_{2}^{2})(1.96+0.842{)}^{2}}{\Delta^{2}}$$ (1)

where, *σ*_1_ and *σ*_2_ are the standard deviation of the outcome measure in the intervention and control groups, respectively, and Δ is the effect size (Δ = *μ*_2_ − *μ*_1_ where *μ*_1_ and *μ*_2_ are the mean of the outcome measure in the trial AED and control group, respectively) [20]. The constants relate to the pre-selection of 80% power and a level of significance of 0.05 (two-sided test). An equal number of patients in each group (*N*/2) was also assumed as per current practice in studies on treatments for neonatal seizures [4, 5, 13, 15, 16].

### Simulations and Analysis

**Fig 2:**
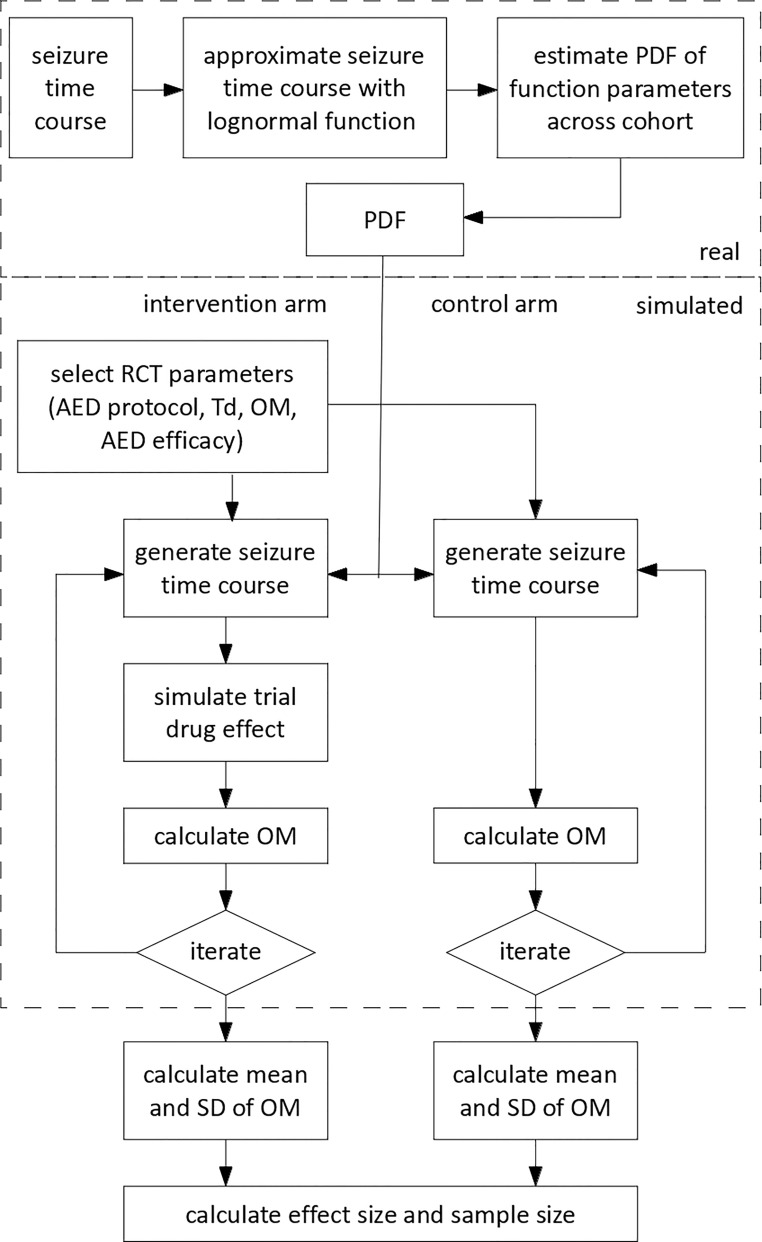
The RCT simulation used in this study. 1) Real seizure burden (SB) time courses were initially modelled with a lognormal function. The probability density function (PDF) of the lognormal function parameters across a cohort of 41 neonates was estimated. 2) RCT parameters including the AED protocol, delay between seizure onset and intervention (T_d_), outcome measure (OM), and level of AED efficacy were selected. A lognormal SB time course for each RCT arm was simulated by selecting lognormal parameters for each arm of the RCT from a multi-variate random variable with the PDF defined previously. The effect of the trial AED (and any other effects according to the AED protocol) was then applied to the SB time course in the intervention arm. The OM was calculated from the SB time course for each RCT arm. This process was iterated across 50000 simulated neonates and the mean and the standard deviation of the OM was then calculated and used to estimate the effect and sample size.

A diagrammatic summary of the process of estimating sample size from simulated RCT is shown in Fig 2. In order to calculate the sample size, the mean and standard deviation of each outcome measure were estimated by simulating 50,000 neonates per group.

We first analysed the control group of several RCT designs in order to estimate how commonly used criteria of treatment success would be met. In these cases, we approximated the control arm of a RCT by selecting the AED protocol, outcome measure and criterion for successful seizure reduction that best matched the definitions from the literature: 1) first line AED, success is an 80% seizure reduction from a 1h period before AED compared to a 1h period after AED; 2) second line AED, success is an 80% seizure reduction from 2h before AED compared to 2h period, 2h after AED; 3) second line AED, success is a 50% seizure reduction from a 1h period before AED compared to a 24h period after AED [4, 12, 13]. We simulated the control group of the RCT for a range of T_d_ (1h to 8h). The proportion of neonates that exceeded the criterion for success was then calculated.

We then analysed the effect of each variable of RCT design independently by simulating subgroups of RCTs where the variable of interest was altered while other variables were fixed. We used a range of T_d_ from 1h to 8h, three AED protocols, four AED efficacies and five outcome measures resulting in 480 simulated trials.

The sample size with respect to each simulated RCT was considered as a random variable. Changes in sample size due to changes in RCT variable were expressed as proportions or fold changes. All values were summarised using the median, interquartile range and range where applicable. The 95% confidence intervals (CI) of the sample and effect sizes were calculated using bootstrap resampling (1000 iterations for each simulated RCT). In this case, the seizure time course of each neonate in the dataset was considered as one sample. Differences in RCT variables (T_d_, outcome measures, AED protocol) were tested using a Wilcoxon signed rank test (a paired test). Finally, we compared the required sample sizes for a subgroup of neonates who had received therapeutic hypothermia to a subgroup who had not received therapeutic hypothermia (normothermic group). This is useful as therapeutic hypothermia has been shown to reduce seizure burden in neonates with HIE and is now the standard of care in many NICUs [19]. Comparisons between therapeutic hypothermia and normothermic groups were performed using Mann Whitney U-tests (an unpaired test).

The trial simulation code was developed with Matlab (Mathworks, Natick, MA, USA) and will be made available on request.

## Results

### Evidence for the need of a control arm

**Fig 3:**
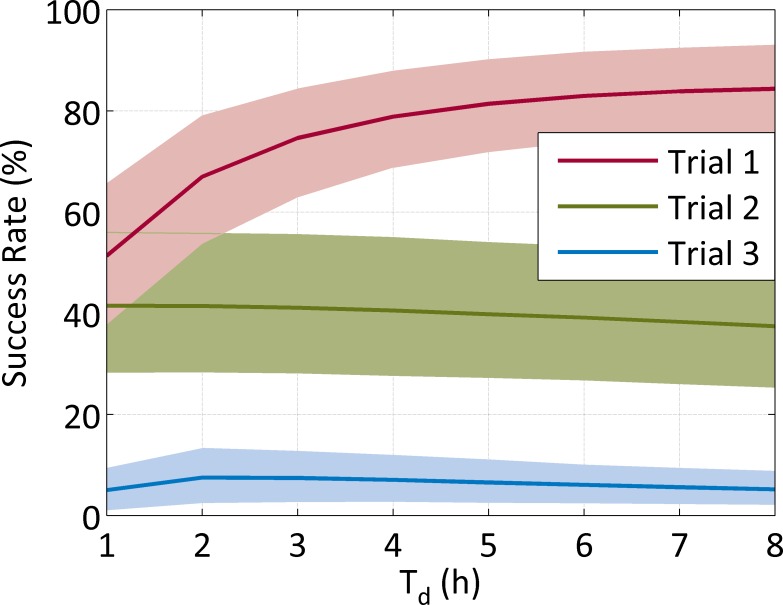
Nominal success rates due to a natural decay in seizure burden in neonates from a placebo control group. Success in trial 1 (second line) 50% reduction in seizure burden measured in a 1h period before placebo compared to the seizure burden in a 24h period after placebo. Success in trial 2 (second line) is an 80% reduction in the seizure burden measured in a 2h period before placebo compared to the seizure burden in a 2h period starting 2h after placebo. Success in trial 3 (first line) is an 80% reduction in the seizure burden measured in a 1h period before placebo compared to the seizure burden in a 1h period after placebo. The shaded areas denote the 95% confidence interval of the estimates.

We found that the placebo control arm of several RCTs had a perceptible rate of apparent success in (Fig 3). This false positive rate was dependent on the definition of the outcome measure, T_d_, and the criterion of successful seizure reduction.

### Sample Size vs T_d_

Tables 1 and 2 show the required sample sizes across RCTs defined by a range of outcome measures, AED protocols and T_d_ for two target levels of AED efficacy: 80% seizure reduction for 12h and 100% seizure reduction for 72h. Tables 3 and 4 shows the corresponding effect sizes. The median increase in sample size from T_d_ = 1h to T_d_ = 8h was 2.1 fold (IQR: 1.7–2.9) across a range of outcome measures, AED protocols and AED efficacies (p<0.001; n = 60).

**Table 1:** The effect of trial design on sample size with an assumed trial drug efficacy of 80% reduction in seizure burden for 12h. Outcome measures (OM) are total seizure burden (tSB) in minutes, post-intervention seizure burden (pSB) in minutes measured over a duration specified by the subscript in hours, seizure burden response (rSB) in minutes per hour, 1h pre-intervention vs. a post-intervention duration specified by the subscript in hours. Results are presented as sample size (95% CI).

AED protocol	OM	Treatment delay (h)				
		1	2	4	6	8
1st line AED	tSB	268 (136–440)	304 (162–508)	422 (236–724)	606 (348–1056)	870 (518–1508)
placebo control	pSB_1_	26(18–34)	32(22–46)	46 (30–70)	58 (36–90)	68 (42–108)
	pSB_12_	42(26–62)	50(30–76)	64 (38–100)	76 (46–120)	88 (52–140)
	rSB_1_	28(18–44)	24(16–36)	32 (20–48)	40 (26–64)	50 (30–80)
	rSB_12_	98(58–182)	102(62–182)	126 (72–234)	152 (86–288)	170 (100–326)
1st line AED	tSB	532(284–902)	610(328–1058)	844 (474–1476)	1206 (708–2138)	1730(1040–3138)
positive control	pSB_1_	608(402–870)	762(538–1190)	1076 (718–1820)	1372 (852–2340)	1654 (992–2860)
	pSB_12_	64(40–100)	72 (44–116)	90 (54–142)	104 (62–166)	118 (70–192)
	rSB_1_	5382 (3028–11158)	7836(4058–17230)	17552 (7004–31878)	23234 (9610–43934)	26444 (10970–67138)
	rSB_12_	194(110–406)	224 (122–460)	316 (162–652)	390 (208–838)	434 (242–924)
2nd line AED	tSB	446(252–770)	516 (294–886)	716 (428–1230)	1016 (616–1764)	1442 (874–2636)
placebo control	pSB_1_	52(32–80)	58 (36–90)	68(42–108)	80 (48–126)	90 (54–144)
	pSB_12_	70(42–110)	76 (46–120)	88(52–140)	98 (58–160)	108 (64–180)
	rSB_1_	36(24–56)	40 (26–64)	50 (30–80)	56 (34–92)	64 (38–106)
	rSB_12_	140(80–266)	152 (86–288)	170 (100–326)	184 (106–350)	196 (110–372)

**Table 2:** The effect of trial design on sample size with an assumed trial drug efficacy of 100% reduction in seizure burden for 72h. Outcome measures (OM) are total seizure burden (tSB) in minutes, post-intervention seizure burden (pSB) in minutes measured over a duration specified by the subscript in hours, seizure burden response (rSB) in minutes per hour, 1h pre-intervention vs. a post-intervention duration specified by the subscript in hours. Results are presented as sample size (95% CI).

AED protocol	OM	Treatment Delay (h)				
		1	2	4	6	8
1st line	tSB	40 (26–60)	46 (28–72)	62 (38–100)	86 (52–146)	118 (72–214)
Placebo	pSB_1_	16 (10–22)	20 (14–28)	28 (18–44)	36 (22–56)	42 (26–68)
Control	pSB_12_	26 (16–40)	32 (20–48)	40 (24–62)	46 (28–74)	54 (32–86)
	rSB_1_	20 (14–30)	22 (14–30)	30 (20–46)	40 (24–62)	48 (30–76)
	rSB_12_	62 (36–112)	70 (42–124)	94 (56–176)	118 (68–220)	134 (80–254)
1st line	tSB	48 (30–76)	54 (34–86)	72 (44–116)	98 (60–172)	138 (82–260)
Positive	pSB_1_	16 (10–22)	20 (14–28)	28 (18–44)	36 (22–56)	42 (26–68)
Control	pSB_12_	34 (20–50)	38 (24–58)	44 (28–72)	52 (32–84)	58 (36–96)
	rSB_1_	252 (176–380)	396 (256–584)	732 (444–1082)	990 (592–1544)	1198 (710–1906)
	rSB_12_	102 (58–200)	128 (72–256)	192 (106–388)	244 (136–482)	278 (158–556)
2nd line	tSB	64 (38–102)	72 (44–116)	96 (58–166)	132 (80–244)	182 (106–370)
Placebo	pSB_1_	32 (20–50)	36 (22–56)	42 (26–68)	50 (30–78)	56 (34–90)
Control	pSB_12_	44 (26–68)	46 (28–74)	54 (32–86)	60 (36–100)	68 (40–112)
	rSB_1_	34 (22–54)	40 (24–62)	48 (30–76)	54 (34–90)	62 (38–102)
	rSB_12_	108 (62–200)	118 (68–220)	134 (80–254)	148 (86–278)	158 (90–300)

**Table 3:** The effect of trial design on effect size with an assumed trial drug efficacy of 80% reduction in seizure burden for 12h. Outcome measures (OM) are total seizure burden (tSB) in minutes, post-intervention seizure burden (pSB) in minutes measured over a duration specified by the subscript in hours, seizure burden response (rSB) in minutes per hour, 1h pre-intervention vs. a post-intervention duration specified by the subscript in hours. The maximum possible effect for each trial is given in the Table 4. The effect size of tSB and pSB_12_ are equal as the assumed efficacy of the trial AED effect is 12h. The effect size of pSB_1_ and rSB_1_ are also equal as the rSB is measured in minutes per hour and not as a proportion. Results are presented as effect size (95% CI).

AED protocol	OM	Treatment delay (h)				
		1	2	4	6	8
1st line AED	tSB & pSB_12_	98.1 (54.2–94.9)	91.3 (48.7–91.4)	77.4 (39.5–81.0)	65.1 (31.5–70.4)	54.9 (25.4–60.5)
placebo control	pSB_1_ & rSB_1_	11.4 (9.2–13.4)	11.3 (8.5–13.6)	10.0 (7.0–12.7)	8.4 (5.6–11.2)	7.0 (4.5–9.7)
	rSB_12_	8.2 (4.5–7.9)	7.6 (4.1–7.6)	6.3 (3.3–6.7)	5.3 (2.6–5.9)	4.5 (2.1–5.0)
1st line AED	tSB & pSB_12_	66.9 (29.9–58.4)	61.3 (27.0–54.9)	51.5 (21.5–47.6)	43.5(17.3–40.9)	36.8 (14.0–35.0)
positive control	pSB_1_ & rSB_1_	0.7 (0.6–0.8)	0.7 (0.5–0.8)	0.6 (0.4–0.8)	0.5 (0.4–0.7)	0.4 (0.3–0.6)
	rSB_12_	5.6 (2.5–4.9)	5.1 (2.2–4.6)	4.2 (1.8–4.0)	3.5 (1.4–3.4)	3.0 (1.2–2.9)
2nd line AED	tSB & pSB_12_	70.9 (35.2–75.3)	64.9 (31.5–70.4)	54.8 (25.4–60.5)	46.5 (20.6–51.8)	39.8 (16.9–44.6)
placebo control	pSB_1_ & rSB_1_	9.2 (6.3–12.0)	8.4 (5.6–11.2)	7.0 (4.5–9.7)	5.9 (3.6–8.4)	4.9 (2.9–7.2)
	rSB_12_	5.8 (2.9–6.3)	5.3 (2.6–5.9)	4.5 (2.1–5.0)	3.8 (1.7–4.3)	3.3 (1.4–3.7)

**Table 4:** The effect of trial design on effect size with an assumed trial drug efficacy of 100% reduction in seizure burden for 72h (the maximum possible effect). Outcome measures (OM) are total seizure burden (tSB) in minutes, post-intervention seizure burden (pSB) in minutes measured over a duration specified by the subscript in hours, seizure burden response (rSB) in minutes per hour, 1h pre-intervention vs. a post-intervention duration specified by the subscript in hours. The effect size of pSB_1_ and rSB_1_ are equal as the rSB is measured in minutes per hour and not as a proportion. Results are presented as effect size (95% CI).

AED protocol	OM	Treatment Delay (h)				
		1	2	4	6	8
1st line	tSB	212.3 (54.2–94.9)	198.1 (48.7–91.4)	170.8 (39.5–81.0)	147.0 (31.5–70.4)	127.0 (25.4–60.5)
Placebo	pSB_1_ & rSB_1_	14.2 (9.2–13.4)	14.1 (8.5–13.6)	12.5 (7.0–12.7)	10.5 (5.6–11.2)	8.8 (4.5–9.7)
Control	pSB_12_	122.7 (54.2–94.9)	114.1 (48.7–91.4)	96.6 (39.5–81.0)	81.1 (31.5–70.4)	68.3 (25.4–60.5)
	rSB_12_	10.2 (4.5–7.9)	9.5 (4.1–7.6)	8.0 (3.3–6.7)	6.7 (2.6–5.9)	5.7 (2.1–5.0)
1st line	tSB	181.0 (29.9–58.4)	168.1 (27.0–54.9)	145.0 (21.5–47.6)	125.3 (17.3–40.9)	108.9 (14.0–35.0)
Positive	pSB_1_ & rSB_1_	3.6 (0.6–0.8)	3.5 (0.5–0.8)	3.1 (0.4–0.8)	2.6 (0.4–0.7)	2.2 (0.3–0.6)
Control	pSB_12_	91.4 (29.9–58.4)	84.2 (27.0–54.9)	70.7 (21.5–47.6)	59.4 (17.3–40.9)	50.2 (14.0–35.0)
	rSB_12_	7.6 (2.5–4.9)	7.0 (2.2–4.6)	5.8 (1.8–4.0)	4.9 (1.4–3.4)	4.1 (1.2–2.9)
2nd line	tSB	158.3 (35.2–75.3)	146.8 (31.5–70.4)	126.8 (25.4–60.5)	110.1 (20.6–51.8)	96.2 (16.9–44.6)
Placebo	pSB_1_ & rSB_1_	11.5 (6.3–12.0)	10.5 (5.6–11.2)	8.8 (4.5–9.7)	7.3 (3.6–8.4)	6.1 (2.9–7.2)
Control	pSB_12_	88.5 (35.2–75.3)	81.1 (31.5–70.4)	68.3 (25.4–60.5)	57.8 (20.6–51.8)	49.3 (16.9–44.6)
	rSB_12_	7.3 (2.9–6.3)	6.7 (2.6–5.9)	5.7 (2.1–5.0)	4.8 (1.7–4.3)	4.1 (1.4–3.7)

### Sample Size vs Outcome Measure

The choice of outcome measure resulted in the largest changes in the required sample size. In general, long term outcome measures resulted in the largest sample sizes and short term measures resulted in the smallest sample sizes. The only exception was for RCTs with a positive control where short term outcome measures had the highest sample size. The choice of outcome measure resulted in a median difference in sample size of, at most, 30.7 fold (IQR: 13.7–40.0) across a range of AED protocols, AED efficacies and delays (p<0.001; n = 96).

### Sample size vs AED protocol

In general, RCTs that included comparisons with a control group containing other AEDs (as either positive controls or first line AEDs in a second line trial) required a higher sample size than first line, placebo controlled trials; the median increase in sample size was 3.2 fold (IQR: 1.9–11.9) across a range of AED efficacies, delays and outcome measures (p<0.001; n = 320).

### Effects on sample size due to therapeutic hypothermia

Required sample sizes were on median 2.6 times greater (IQR: 2.4–3.0) for a RCT based on seizure burden time courses from a subgroup of neonates treated with hypothermia compared to normothermic neonates (p<0.001; n = 480). More detailed results from the analysis of a subgroup of neonates treated with therapeutic hypothermia is shown in S3 Appendix.

## Discussion

Our results suggest that the design of a RCT for assessing the efficacy of an AED in neonates with seizures is challenging with orders of magnitude differences in the required sample size possible depending on the choices of AED protocol, outcome measure, and T_d_. The high variability in the magnitude of seizure burden over time within and across neonates translates to high variability in outcome measures of trial AED efficacy. The present findings offer three practical suggestions for how to optimize a study in terms of minimizing the required sample size and maximizing the validity.

*Trials must incorporate a control group*. A control group is necessary as simulations show a non-negligible percentage (5–85%) of patients treated with a placebo can fulfil criteria of treatment success due to the natural reduction in seizures over time. This is commonly seen in drug trials for epilepsy [2]. Indeed, it is plausible to speculate that this natural seizure reduction may contribute, in varying degrees, to the reported treatment effects observed in prior uncontrolled studies [13, 21, 22].

*Trials should aim to minimise*, *and control for differences in*, *T*_*d*_ *between intervention and control groups*. A reduction in T_d_ reduces the required sample size and increases the measured effect. A change in T_d_ of several hours can double the required sample size. This implies that the entire study protocol needs to proceed very rapidly, advancing from patient identification to recruitment and AED administration within a few hours of seizure onset. This is technically achievable in high level NICUs as modern clinical practice can operate within short time frames as long as early EEG monitoring of all high-risk infants is standard procedure [23, 24]. The dependency of the effect size on T_d_ indicates that any differences in T_d_ between intervention and control groups must be controlled for in statistical analyses.

*The combination of AED protocol and the choice of outcome measure should be carefully considered as a mismatch may result in an order of magnitude increase in the required sample size*. Among outcome measures and AED protocols, post-intervention seizure burden in a first line AED vs. placebo controlled RCT required the lowest required sample size. RCTs using short-term seizure burden response in a positive control trial had the highest required sample size. For other AED protocols, RCTs with the total seizure burden as an outcome measure required large sample sizes. The post-intervention seizure burden was, in general, the outcome measure that resulted in RCTs with the lowest required sample size depending on the post-intervention analysis period. The ability to alter the post-intervention period is advantageous as investigators can nominate a desired trial AED effect time which caters for the incorporation of rescue medications in trial design for seizures that do not respond to treatment or reoccur with significant intensity; medications that if given during the analysis period confound subsequent statistical analyses. Another advantage of the post-intervention seizure burden is that it does not require the monitoring of a baseline period (seizure burden response) or the recording of seizure onset (total seizure burden).

Our present findings are supported by the literature both in terms of studies that did, and did not find, significant differences in seizure burden. In the highly cited and important study of Painter et al. (1999), no significant differences in seizure burden response between phenobarbitone and phenytoin were found in a cohort of 59 neonates [4]. Our findings suggest that this trial was powered to detect differences in seizure efficacy considerably larger than 25% between AEDs (see positive control, first line, rSB_1_ in Table 2 – 75% efficacy for positive control vs 100% efficacy for trial drug); in other words, the efficacy of phenobarbitone was not greater than 25% different from phenytoin. A recent uncontrolled study observed AED efficacy with a measure similar to the seizure burden response in a cohort of 19 neonates [17]. In an RCT with a similar design and effect size (placebo control, first line, rSB_1_, T_d_ = 1h in Table 1); our model would have predicted a sample size of 14 neonates per group. Low et al. (2012) also showed a significant reduction in total seizure burden in neonates treated with hypothermia in a cohort of 31 neonates [19]. In this case, our model would predict a cohort of 40 neonates based on a similar effect size; equivalent to an AED effect of 100% for 72h given within 1h of seizure onset (see placebo control, first line, TSB in Table 2). The use of simulation adds further context to these studies which can be useful for clinicians who are considering altering their treatment protocols in response to RCT findings.

RCTs may be able to reduce the required sample size by assuming more conservative levels of AED efficacy and considering post-hoc stratification of patients according to the intensity of seizure burden. This approach was used successfully, in a recent study that initially found no significant reduction in total seizure burden due to AED treatment managed with EEG monitoring in a cohort of 35 neonates despite a large effect size; however, a significant reduction in total seizure burden was found in a subgroup of neonates with seizures but without status epilepticus [16].

A potential limitation in our work is that only one aetiology (HIE) of neonatal seizures was used as the basis of our simulations. While systematic comparative characterization of seizure time courses is not available across different aetiologies, the present data and clinical experience suggest that seizure time courses may vary with aetiology [9]. This implies that our sample size estimates apply to treatment trials only on neonates with HIE. If sample size estimates for a general cohort of neonates with seizure are required then the use of neonates with HIE provides the best initial data as 1) HIE is the most common aetiology of seizure and 2) the temporal evolution of seizures over time is well defined in this aetiology [6, 7, 10, 25]. The seizures used as a basis for simulations are detected using the visual interpretation of the multi-channel EEG [23]. While this method is the gold standard for seizure detection, it is not perfect and its reliability is reduced when seizures are infrequent or of short duration [26]. We do not expect this to have had a major effect on the results as any variability due to reliability will be vastly outweighed by the variability in seizure time courses across neonates. A technical limitation of our work is the use of simulated seizure burden time courses based on a real but limited dataset of neonates where seizures were treated with AEDs. This reduces the precision of the sample size estimates, however, the relationships between outcome measure, trial protocol, treatment delay, level of trial AED efficacy and sample size are statistically significant.

Our present work shows that it is, in principle, possible to measure the short term efficacy of AEDs in a relatively small cohort of neonates. A more difficult challenge is determining if one AED is more effective than another (RCTs with a positive control); a proposition that more closely achieves clinical equipoise [27]. In order to minimise the sample size, these RCTs require long term outcome measures of seizure burden and must assume a high level of trial AED efficacy far in excess of the current generation of AEDs (see Table 2). This assumption is more critical as modern day NICUs are highly effective positive controls that take advantage of EEG monitoring to target AED therapy and use therapeutic hypothermia for HIE which have, independently, been shown to significantly reduce seizure burden [16, 19].

## Supporting Information

S1 Appendix A summary of the seizure burden in the cohort used to construct a model of seizure time courses. (DOCX)

S2 Appendix A model of seizure time courses and trial simulation. (DOCX)

S3 Appendix Additional results based on alternate simulations parameters. (DOCX)
