# Supplementary material for: Treatment Trials for Neonatal Seizures: The Effect of Design on Sample Size
Source: PLoS One. 2016 Nov 8;11(11):e0165693. doi: 10.1371/journal.pone.0165693 (PMC5100925; doi:10.1371/journal.pone.0165693)
Supplement: S1 Appendix — (DOCX) [file pone.0165693.s001.docx]

**Appendix S1:** *A summary of the seizure burden (SB) in the cohort used to construct a model of seizure time courses and the calculation of seizure burden.*

**Table S1:** Temporal characteristics of electrographic seizures in neonates used to simulate seizure time courses (*n*=41). * with respect to seizure onset.

|  | Median | IQR |
| --- | --- | --- |
| Seizure Period (hours) | 27.8 | 11.3-48.3 |
| Total Seizure Burden (mins) | 118.2 | 43.1-227.8 |
| Total Seizure Number | 46 | 16-128 |
| Seizure Burden (mins/hr) | 5.4 | 3.4-9.9 |
| Mean Seizure Duration (s) | 142 | 92-300 |
| Number of Seizures (per hr) | 2.2 | 1.2-3.4 |
| Maximum Seizure Burden (per hour) | 27.7 | 16.3-41.8 |
| Time of Maximum Seizure Burden (hours)* | 3.3 | 0.5-8.3 |

The presence or absence of a seizures is annotated by the human expert using visual interpretation of the EEG. In this case, a time series is generated with a sample every one second where 1 denotes the presence of seizure and 0 denotes the absence of seizure or non-seizure. This can be defined as,

$$s\left( t \right)=\left\{ \begin{matrix} 0 & \text{when seizure is absent} \\ 1 & \text{when seizure is present} \end{matrix} \right.$$

In order to calculate the seizure burden over time, the following equation is used,

$$s_{b}\left( \tau\right)=\int_{\tau-T}^{\tau+T} s\left( t \right) dt$$

where, *τ* and *t* are time, and *T* is half the window duration. An example calculation is show in Figure 1.


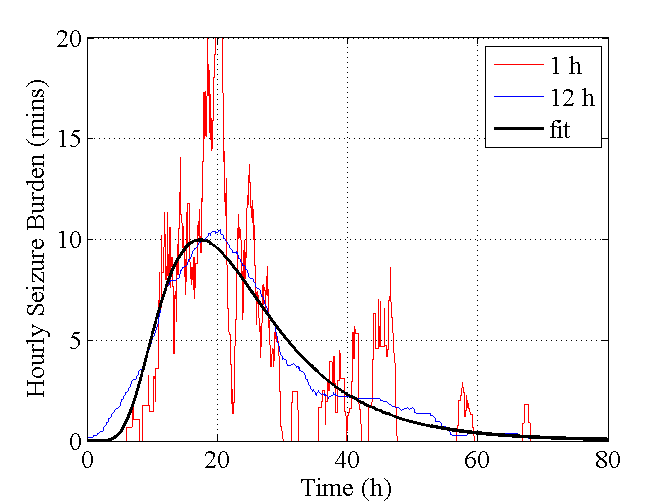


**Figure S1.1:** The calculation of seizure burden over time using different time periods for the calculation. The red line is calculated using a 1h window, the blue line is calculated using a 12h window and the black line is a lognormal function fit to the data.
