# Supplementary material for: Treatment Trials for Neonatal Seizures: The Effect of Design on Sample Size
Source: PLoS One. 2016 Nov 8;11(11):e0165693. doi: 10.1371/journal.pone.0165693 (PMC5100925; doi:10.1371/journal.pone.0165693)
Supplement: S2 Appendix — (DOCX) [file pone.0165693.s002.docx]

**Appendix S2:** *A Model of Seizure Time Courses and Trial Simulation*

*Simulating SB time courses*: The lognormal function was used to generate a seizure burden time course. It is defined as,

$s\left( t; \lambda\right)=\alpha\text{exp}\left( -\frac{\left( ln(t)-\mu\right)^{2}}{{2\sigma}^{2}} \right), t>0$ (1)

In order to estimate the distribution of $\lambda=[\mu,\sigma,\alpha]$, this function was fit to SB time courses recorded from 41 neonates with hypoxic ischaemic encephalopathy. Details on the cohorts that make up this cohort can be found in Lynch et al. (2012 and 2015).^7, 10^

A least squares fit was used and the distribution of the dataset was approximated with a Gaussian mixture model with 2 mixtures. The parameters for these two mixtures were,

$$m_{1}=\left[ \begin{matrix} 1.67 & 0.79 & 47.40 \end{matrix} \right]$$

$$s_{1}=\left[ \begin{matrix} \begin{matrix} 0.94 & -0.43 & -30.54 \end{matrix} \\ \begin{matrix} -0.43 & 0.29 & 18.27 \end{matrix} \\ \begin{matrix} -30.54 & 18.27 & 1706.47 \end{matrix} \end{matrix} \right]$$

$$m_{2}=\left[ \begin{matrix} 0.50 & 0.59 & 22.55 \end{matrix} \right]$$

$$s_{2}=\left[ \begin{matrix} \begin{matrix} 0.97 & -0.11 & -8.97 \end{matrix} \\ \begin{matrix} -0.11 & 0.07 & 0.19 \end{matrix} \\ \begin{matrix} -8.97 & 0.19 & 223.57 \end{matrix} \end{matrix} \right]$$

For $\lambda=[\mu,\sigma,\alpha]$, where $m$ is the mean of $\lambda$, and $s$ is the covariance of $\lambda$. The ratio of mixture 1 to 2 was approximately 1:1 (22:19).

*Model assessment:* The differences between the real cohort and a simulated cohort based on the actual fit were small. The median difference in total SB was -0.8 minutes (IQR: -5.5 to 10.3 minutes; p=0.53 Wilcoxon Sign Rank Test), in maximum SB was 6.5 minutes per hour (IQR: -1.3 to 10.6 minutes per hour; p<0.001 Wilcoxon Sign Rank Test) and in the time of maximum SB was 0.4h (IQR: -0.2-1.3h; p=0.07 Wilcoxon Sign Rank Test). The only significant difference between real and simulated seizure time courses was a lower maximum SB due to the smoothing of the lognormal function.

*Trial simulation:* A SB time course of 72h in length was simulated using (1) where $\lambda$ was drawn from a multivariate Normal distribution. For every SB time course drawn from mixture one, 0.86 time courses were drawn from mixture 2. The generated SB time course was then examined and included in the simulated cohort if 1) the total SB was greater than 5 minutes and 2) the maximum SB was less than 60 minutes per hour.

The SB time courses for a simulated first line, placebo control trial were then defined as,

$$s_{n}^{1}\left( t \right)=s_{a}\left( t \right),$$

$$s_{i}^{1}\left( t \right)=\left\{ \begin{matrix} s_{b}\left( t \right) & 0<t\leq T_{d} \\ Ds_{b}\left( t \right) & T_{d}<t<T_{d}+T_{m} \end{matrix}, \right.$$

where *D* denotes the assumed reduction (e.g. *D* = 0.5 or 0.2 for a 50% or 80% reduction, respectively), *T_m_* is the duration of the effect in hours, the superscript denotes the trial type (1^st^ line or 2^nd^ line), the subscript on the left hand side of the equation denotes the time course, *s(t)* (*n*, no intervention arm or *i*, intervention arm), the subscript on the right hand side of the equation (*a* or *b*) differentiates different realizations of the underlying seizure time course, *T_d_* is the time of drug administration.

When simulating a trial of a first line, positive control trial, the following SB time courses were used.

$$s_{n}^{1}\left( t \right)=\left\{ \begin{aligned} \begin{matrix} s_{a}(t) & 0<t\leq T_{d} \end{matrix} \\ \begin{matrix} {0.25s}_{a}(t) & 0<t\leq T_{d}+3 \end{matrix} \\ \begin{matrix} s_{a}(t) & T_{d}+3<t\leq T_{\text{off}} \end{matrix} \end{aligned} \right.$$

$$s_{i}^{1}\left( t \right)=\left\{ \begin{matrix} s_{b}\left( t \right) & 0<t\leq T_{d} \\ Ds_{b}\left( t \right) & T_{d}<t< T_{d}+T_{m} \end{matrix}, \right.$$

For the case where the intervention was a second line treatment, we assumed an immediate fixed effect of a 75% reduction that lasted for 3h. This was based on our recent work analysing the effectiveness of phenobarbitone as a first line AED (Low et al., 2016).^17^ Placebo was assumed to have no effect.

$$s_{p}^{2}\left( t \right)=\left\{ \begin{aligned} \begin{matrix} s_{a}\left( t \right) & 0<t{\leq T}_{1} \\ 0.25s_{a}\left( t \right) & T_{1}<t{\leq T}_{1}+3 \\ s_{a}\left( t \right) & T_{1}+3<t{\leq T}_{d} \end{matrix} \\ \begin{matrix} s_{a}\left( t \right) & T_{d}<t{\leq T}_{\text{off}} \end{matrix} \end{aligned} \right.$$

$$s_{t}^{2}\left( t \right)=\left\{ \begin{aligned} \begin{matrix} s_{b}(t) & 0<t{\leq T}_{1} \end{matrix} \\ \begin{matrix} {0.25s}_{b}(t) & T_{1}<t{\leq T}_{1}+3 \end{matrix} \\ \begin{matrix} s_{b}(t) & T_{1}+3<t{\leq T}_{d} \end{matrix} \\ \begin{matrix} {Ds}_{b}(t) & T_{d}<t{\leq T_{d}+T_{m}} \end{matrix} \end{aligned} \right.$$

In this case, *T*_1_ is the time of the administration of the first line AED.

Outcome measures were then defined as,

$$\text{total SB}=\int_{0}^{T} s\left( t \right) dt$$

$$\text{post-intervention SB}=\int_{T_{d}}^{T_{w}} s\left( t \right) dt$$

$$\text{SB response}=\frac{1}{T_{w}-T_{d}}\int_{T_{d}}^{T_{w}} s\left( t \right) dt-\frac{1}{T_{d}-T_{a}}\int_{T_{a}}^{T_{d}} s\left( t \right) dt$$

For these outcome measures the time periods of analysis can be adjusted and *T* is a time point that denotes the end of monitoring or seizure offset, *T_w_* is a user defined time that must be after *T_d_*, *T_d_* is the time of intervention, and *T*_a_ is a user defined time before *T_d_*.
