# Supplementary material for: Treatment Trials for Neonatal Seizures: The Effect of Design on Sample Size
Source: PLoS One. 2016 Nov 8;11(11):e0165693. doi: 10.1371/journal.pone.0165693 (PMC5100925; doi:10.1371/journal.pone.0165693)
Supplement: S3 Appendix — (DOCX) [file pone.0165693.s003.docx]

**Appendix S3:** *Additional results based on alternate simulations parameters.*

**Table S3.1:** Trial simulations based on a subgroup of neonates who were treated with therapeutic hypothermia (n=23); estimated samples sizes for different delays in intervention, outcome measures and AED protocols. The assumed trial AED effect is an 80% reduction in seizures for 12h. Interventions include phenobarbitone (incumbent AED/positive control), trial drug and a placebo. Outcome measures (OM): tSB – total seizure burden (mins), pSB – post intervention seizure burden (mins) – the subscript denotes the length of the window in hours analysed, rSB – seizure burden response (mins/h), 1h pre-intervention vs. the post-intervention duration given in the subscript. Sample sizes are presented as sample size (95% confidence interval).

|  |  | Treatment Delay (h) | | | | | | | |
| --- | --- | --- | --- | --- | --- | --- | --- | --- | --- |
|  | OM | 1 | 2 | 3 | 4 | 5 | 6 | 7 | 8 |
| 1st line | tSB | 286 (136-696) | 360 (190-832) | 456 (254-1018) | 578 (338-1252) | 730 (450-1574) | 918 (566-1972) | 1150 (710-2516) | 1430 (868-3090) |
| Placebo | pSB_1_ | 30 (14-38) | 42 (18-52) | 54 (22-68) | 64 (26-86) | 74 (30-104) | 84 (36-124) | 92 (42-144) | 98 (46-166) |
| Control | pSB_12_ | 56 (24-64) | 70 (30-90) | 80 (36-116) | 90 (42-140) | 100 (50-166) | 108 (56-194) | 116 (62-224) | 122 (68-252) |
|  | rSB_1_ | 38 (16-54) | 36 (16-56) | 40 (16-62) | 46 (20-72) | 54 (22-88) | 62 (26-102) | 68 (30-116) | 74 (32-130) |
|  | rSB_12_ | 172 (76-408) | 190 (86-522) | 200 (84-540) | 218 (86-574) | 238 (88-598) | 256 (92-626) | 272 (96-652) | 284 (102-672) |
| 1st line | tSB | 648 (400-1700) | 788 (496-2028) | 978 (622-2470) | 1230 (798-3068) | 1550 (988-3790) | 1950 (1216-4688) | 2442 (1444-5828) | 3042 (1706-7392) |
| Positive | pSB_1_ | 740 (354-950) | 1028 (410-1332) | 1342 (508-1814) | 1626 (614-2380) | 1872 (728-2924) | 2090 (842-3442) | 2292 (964-4016) | 2482 (1072-4594) |
| Control | pSB_12_ | 94 (40-136) | 108 (48-168) | 120 (56-200) | 130 (64-234) | 140 (72-266) | 148 (80-302) | 158 (86-336) | 166 (92-374) |
|  | rSB_1_ | 6320 (2986-14426) | 12208 (4456-30374) | 17278 (5288-47126) | 20320 (6302-69692) | 23406 (7334-91374) | 26764 (8422-148420) | 30088 (9346-190966) | 33198 (10316-269884) |
|  | rSB_12_ | 400 (168-1400) | 486 (202-1736) | 536 (202-1840) | 590 (208-1906) | 644 (214-1930) | 690 (226-1942) | 728 (236-1982) | 754 (246-2014) |
| 2nd line | tSB | 602 (398-1468) | 728 (490-1768) | 896 (610-2140) | 1116 (740-2630) | 1394 (888-3194) | 1734 (1048-3950) | 2148 (1228-4860) | 2646 (1440-6154) |
| Placebo | pSB_1_ | 74 (30-104) | 84 (36-124) | 92 (42-144) | 98 (46-166) | 106 (52-190) | 112 (58-214) | 120 (62-238) | 126 (68-264) |
| Control | pSB_12_ | 100 (50-166) | 108 (56-194) | 116 (62-224) | 122 (68-252) | 128 (74-280) | 136 (80-312) | 142 (86-342) | 148 (90-372) |
|  | rSB_1_ | 54 (22-88) | 62 (26-102) | 68 (30-116) | 74 (32-130) | 80 (36-146) | 84 (40-162) | 90 (44-180) | 94 (48-196) |
|  | rSB_12_ | 238 (88-598) | 256 (92-626) | 272 (96-652) | 284 (102-672) | 292 (108-690) | 300 (112-712) | 306 (118-732) | 310 (124-752) |

**Table S3.2:** Trial simulations based on a subgroup of neonates who were treated with therapeutic hypothermia (n=23); estimated effect sizes for different delays in intervention, outcome measures and AED protocols. The assumed trial AED effect is an 80% reduction in seizures for 12h. Interventions include phenobarbitone (incumbent AED/positive control), trial drug and a placebo. Outcome measures (OM): tSB – total seizure burden (mins), pSB – post intervention seizure burden (mins) – the subscript denotes the length of the window in hours analysed, rSB – seizure burden response (mins/h), 1h pre-intervention vs. the post-intervention duration given in the subscript. The effect size of tSB and pSB_12_ are equal as the assumed efficacy of the trial AED effect is only 12h. Effect sizes are presented as effect size (95% confidence interval).

|  |  | Treatment Delay (h) | | | | | | | |
| --- | --- | --- | --- | --- | --- | --- | --- | --- | --- |
|  | OM | 1 | 2 | 3 | 4 | 5 | 6 | 7 | 8 |
| 1st line | tSB & pSB_12_ | 62.1 (54.2-94.9) | 55.2 (48.7-91.4) | 49.2 (43.8-86.1) | 44.0 (39.5-81.0) | 39.5 (35.2-75.3) | 35.5 (31.5-70.4) | 32.0 (28.3-65.4) | 28.9 (25.4-60.5) |
| Placebo | pSB_1_ & rSB_1_ | 9.3 (9.2-13.4) | 8.2 (8.5-13.6) | 7.2 (7.7-13.2) | 6.4 (7.0-12.7) | 5.7 (6.3-12.0) | 5.0 (5.6-11.2) | 4.5 (5.0-10.4) | 4.0 (4.5-9.7) |
| Control | rSB_12_ | 5.3 (4.5-7.9) | 4.6 (4.1-7.6) | 4.1 (3.6-7.2) | 3.7 (3.3-6.7) | 3.3 (2.9-6.3) | 3.0 (2.6-5.9) | 2.7 (2.4-5.4) | 2.5 (2.1-5.0) |
| 1st line | tSB & pSB_12_ | 38.9 (29.9-58.4) | 34.8 (27.0-54.9) | 31.2 (24.1-51.0) | 28.0 (21.5-47.6) | 25.2 (19.3-44.1) | 22.8 (17.3-40.9) | 20.6 (15.7-37.8) | 18.7 (14.0-35.0) |
| Positive | pSB_1_ & rSB_1_ | 0.6 (0.6-0.8) | 0.5 (0.5-0.8) | 0.4 (0.5-0.8) | 0.4 (0.4-0.8) | 0.4 (0.4-0.8) | 0.3 (0.4-0.7) | 0.3 (0.3-0.7) | 0.3 (0.3-0.6) |
| Control | rSB_12_ | 3.4 (2.5-4.9) | 2.9 (2.2-4.6) | 2.6 (2.0-4.2) | 2.4 (1.8-4.0) | 2.2 (1.6-3.7) | 2.0 (1.4-3.4) | 1.8 (1.3-3.2) | 1.6 (1.2-2.9) |
| 2nd line | tSB & pSB_12_ | 39.4 (35.2-75.3) | 35.5 (31.5-70.4) | 32.0 (28.3-65.4) | 28.9 (25.4-60.5) | 26.2 (22.9-56.0) | 23.9 (20.6-51.8) | 21.8 (18.6-48.0) | 19.9 (16.9-44.6) |
| Placebo | pSB_1_ & rSB_1_ | 5.7 (6.3-12.0) | 5.0 (5.6-11.2) | 4.5 (5.0-10.4) | 4.0 (4.5-9.7) | 3.6 (4.0-9.0) | 3.2 (3.6-8.4) | 2.9 (3.2-7.7) | 2.6 (2.9-7.2) |
| Control | rSB_12_ | 3.3 (2.9-6.3) | 3.0 (2.6-5.9) | 2.7 (2.4-5.4) | 2.5 (2.1-5.0) | 2.2 (1.9-4.7) | 2.0 (1.7-4.3) | 1.9 (1.5-4.0) | 1.7 (1.4-3.7) |
